# Artificial Neural Network Prediction of Mortality in Cancer Patients Presenting for Radiation Therapy at a Multisite Institution

**DOI:** 10.7759/cureus.64536

**Published:** 2024-07-14

**Authors:** Elan Shahrabani, Michael Shen, Yen-Ruh Wuu, Louis Potters, Bhupesh Parashar

**Affiliations:** 1 Department of Radiation Oncology, Northwell/Donald and Barbara Zucker School of Medicine at Hofstra, New Hyde Park, USA

**Keywords:** ai, prediction, mortality, oncology, radiation, machine learning, artificial intelligence

## Abstract

Introduction: For many decades, the management of cancer has utilized radiation therapy, which continues to evolve with technology to improve patient outcomes. However, despite the standardization of treatment plans and the establishment of best clinical practices based on prospective, randomized trials and adherence to National Comprehensive Cancer Network (NCCN) guidelines, the outcomes from radiation therapy are highly variable and dependent on a number of factors, including patient demographics, tumor characteristics/histology, and treatment parameters. In this study, we attempt to use available patient data and treatment parameters at the time of radiation therapy to predict future outcomes using artificial intelligence (AI).

Methods: Six thousand five hundred ninety-five cases of patients who completed radiation treatment were selected retrospectively and used to train artificial neural networks (ANNs) and baseline models (i.e., logistic regression, random forest, support vector machines [SVMs], gradient boosting [XGBoost]) for binary classification of mortality at multiple time points ranging from six months to five years post-treatment. A hyperparameter grid search was used to identify the optimal network architecture for each time point, using sensitivity as the primary outcome metric.

Results: The median age was 75 years (range: 2-102 years). There were 63.8% females and 36.1% males. The results indicate that ANNs were able to successfully perform binary mortality prediction with an accuracy greater than random chance and greater sensitivity than baseline models used. The best-performing algorithm was the ANN, which achieved a sensitivity of 83.00% ± 4.89% for five-year mortality.

Conclusion: The neural network was able to achieve higher sensitivity than Logistic Regression, SVM Random Forest, and XGBoost across all output target variables, demonstrating the utility of a neural network model for mortality prediction on the provided dataset.

## Introduction

Accurate prediction of mortality in cancer patients is important in deciding the optimal management strategy and individualized treatment plan while taking into consideration quality of life. At present, most management decisions are based on patient age/functional status, tumor characteristics, and standardized treatment guidelines formulated by the National Comprehensive Cancer Network (NCCN) and ASTRO, which are based on scientific evidence such as phase I-III trials. Present prognostic systems use TNM staging, which incorporates a limited number of histopathologic variables (i.e., tumor size, degree of regional lymph node involvement, and presence of distant metastasis), to predict patient survival over a specified period of time. However, these systems stratify the population of cancer patients into extremely broad categories and thus cannot provide a more accurate projected outcome that incorporates all relevant prognostic data in a patient’s record [1]. In order to improve upon our ability to more accurately predict patient outcomes, additional patient variables must be taken into consideration. Consequently, there is potential for continued growth with the incorporation of artificial intelligence (AI) and machine learning (ML) for prognostication.

AI/ML algorithms can explore large datasets with many inputs, identify complex patterns and relationships between multiple variables, and generate models to predict outcomes. ML has progressed significantly since Arthur Samuel first coined the term in the 1950s. ML is divided into supervised learning, unsupervised learning, and reinforcement learning [2]. Supervised learning is a subcategory of ML and AI, defined by its use of labeled datasets with inputs and correct outputs to train algorithms to classify data or predict outcomes accurately. In comparison, unsupervised learning algorithms infer patterns from a dataset without reference to known or labeled outcomes.

Several commonly used supervised machine learning models will be described briefly. Logistic regression is a technique that uses a logistic model with multivariate input and outputs a binary value (i.e., 1 or 0). It performs computations comparable to those of a single-node neural network. Random forest is a machine learning technique that uses an ensemble of individual decision trees, with each “tree” outputting a binary output, with the most common output being the consensus decision. A support vector machine (SVM), also known as a support vector classifier (SVC), is a non-probabilistic binary linear model commonly used in classification problems that sort data into two groups by maximally separating the data points of different classes in distinct regions of space. It can handle linearly as well as non-linearly separable data. Xtreme gradient boost (XGBoost) is another method using classification trees and is popular due to its scalability and high accuracy [3-5].

Artificial neural networks (ANNs) are a class of statistical learning models that have demonstrated success in the domains of image and speech recognition and have recently become widespread in processing biomedical data [6]. An ANN is composed of computational nodes ("neurons"), which each take a weighted summation over a series of inputs and pass that sum through a nonlinear transfer function (e.g., the sigmoid function) to produce an output. Multiple neurons are then assembled in layers, whose sequential outputs are passed to each other until a terminal output node or nodes are reached. The advantage of an ANN is its ability to produce a complex, nonlinear representation of a large number of input variables for use in classification or regression problems [7]. 

One study showed that ANNs incorporating the type of radiation treatment as input were able to predict mortality in patients who received radiotherapy for breast cancer [8]. Radiation therapy (RT) is a mainstay of cancer treatment and is used for both palliative and curative measures. Radiation treatment decisions, including radiation dose, fractionation, and treatment intent, are based on established standards of care regimens, the tolerability of treatment, potential acute and chronic side effects, and the impact on quality of life. An interesting question is whether the fine-tuned parameters of radiation therapy delivery and patient demographics can be used to augment a predictive model of patient mortality. This study aims to evaluate the efficacy of ANNs in predicting six-month, one-year, three-year, and five-year mortality in a dataset of patients undergoing radiation therapy for various malignancy types. Throughout this study, we employ several machine learning techniques to create baseline models against which ANN performance is compared. 

## Materials and methods

Institutional review board (IRB) approval was obtained to collect individual patient data from institutional electronic medical records. We collaborated with ONCORA, an integrated system that extracts data from electronic medical records across our institutional network. Patient cases were selected from the data source using the following criteria: primary cancers of the prostate, breast, gynecological, thoracic, gastrointestinal, spine, and brain. The dataset included both cases of palliative and curative intent (stages I-IV). Appendix A enumerates the data files collected. 

Clinically, KPS (Karnofsky Performance Status) is a critical variable for assessing prognosis. During the initial dataset analysis, we discovered 2407 missing values for the KPS score. Therefore, we categorized patients into two separate datasets: the KPS dataset (patients with KPS) or the NKPS dataset (all patients with the KPS variable removed), which captures all cases of the original dataset. We organized the data in such a fashion because predictive models expect non-empty values for each variable.

The code written for this study was developed and tested on three separate computers: (i) an i7-3520M 2.90 GHz CPU and no dedicated graphics card (GPU); (ii) an i7-4790 3.60 GHz CPU and Nvidia GeForce GTX 745 GPU; and (iii) an i7-8750H CPU and Nvidia GeForce GTX 1070. The environments used to test the runnability of this study were Ubuntu 18.04 (64-bit), Windows 10 Home (64-bit, v.1809), Windows 10 Pro (64-bit, v.1809), Windows 10 Pro (64-bit, v.1903), Windows Subsystem for Linux running Ubuntu 18.04 (64-bit), and macOS Mojave 10.14.6. Python 3 was used to write the code for this study. The version of Keras used was 2.2.4, with underlying TensorFlow 1.14.0 (CPU and GPU packages). The version of Pandas used was 0.24.2. The version of NumPy used was 1.16.4 (https://numpy.org/). The version of Scikit-Learn used was 0.21.2 [9-13].

Table 1 includes a summary of the ML algorithms used in this study, including the advantages and disadvantages of each.

**Table 1:** A summary of machine learning algorithms used in this study.

ML Algorithm	Advantages	Disadvantages
Logistic Regression	Easier to implement and interpret, fast training time, intuitive probabilistic view of prediction	Lacks flexibility in capturing complex relationships and may overfit when number of features is large
SVM	Works well when there is a clear margin of separation within data, effective in high-dimensional spaces	Less effective with large datasets, less robust against noise, and does not provide an estimate of probability directly
Random Forest	Ensemble method works well with missing data	Computationally expensive
Gradient Boosting	Ensemble method works well with missing data	Computationally expensive
ANN	Ability to learn complex non-linear relationships, generalize well, better in dealing with unstructured input	Computationally expensive, Black box nature of model interpretation

Parameter selection 

The starting parameters for the model included sex, race, cancer site, treatment site, stage, cancer histology, recurrence, attending physician, diagnosis type, disease system, treatment intent, treatment system, age at first RT fraction, weight, religion, marital status, KPS score, Eastern Oncology Cooperative Group (ECOG) grade, TNM stage, treatment energy, treatment modality, total dose, total fractions, dose per fraction, elapsed treatment days, toxicities by grade, and complications including dysphagia, nausea, depression, and dermatitis. A complete enumeration of raw data variables and a dictionary of possible values for the categorical variables are provided in Appendices B and C.

The following variables were excluded: case_id, attending_physician, date_first_fraction, cb_death, date_of_death, main_diagnosis_text, primary_diagnosis_text, secondary_diagnosis_text. Case ID would have no predictive value due to being an artifact of the electronic health record. We chose not to include the physician, even though this may have an impact on outcomes due to the large number of providers. Any variables that would proactively impart death information, including time of death and complications of treatment by death, were dropped due to the target variable of interest being mortality. Freetext variables were not included since they would not be able to be encoded to serve as useful model inputs. In addition, we dropped kps_score in the NKPS dataset only for the reasons described above.

Data preprocessing 

The raw data underwent a cleaning and preprocessing step in Python. The possible target variables in the dataset were mortality within six months, one year, three years, and five years. In the second step, preprocessing of the data was performed. This step consisted of one-hot encoding, binarization of the target, and minimum-maximum scaling. We used a Pandas built-in function to perform one-hot encoding of all categorical variables. Using Scikit-Learn (a machine learning library for Python programming), a minimum-maximum scaler was fitted to the continuous variables of the data and was used to scale all continuous variables to a range of 0 to 1.

In our neural networks, we did not manually adjust the parameters of the networks. Instead, almost all hyperparameters, as well as the number of neurons in the hidden layers, were grid-searched using Scikit-Learn to determine the optimal network design. We configured the network to use either one or two hidden layers with variable numbers of neurons in each, depending on the grid search parameters. The full training and evaluation processes described here were repeated for each of the four mortality variables for both datasets (KPS and NKPS), totaling eight self-contained experiments. Internal to the grid search algorithm, training vectors of cancer cases were passed to a Keras neural network. Keras used its TensorFlow engine backend to compute the output at the final neuron and used the Adam optimizer to update the weights of the network to minimize the loss function, binary cross-entropy.

GridSearchCV’s algorithm created a neural network for each set of 54 parameters, performed training using Keras, and finally ran three-fold cross-validation across several metrics: binary accuracy, sensitivity, specificity, positive predictive value (PPV), and negative predictive value (NPV). Each metric was outputted with an average and standard deviation for that network combination. In order to obtain a baseline for comparison, other classification algorithms were applied to the data: logistic regression, random forest, and SVM, as implemented in Python’s Scikit-Learn library; and extreme gradient boosted trees, as implemented in Python’s XGBoost module.

The models were constructed with default library parameters, except the random forest classifier, which was created with 1000 classification trees. The datasets used for this section were the NKPS and KPS datasets, as described previously. Training and evaluation were performed using an 80:20 split of training to testing data ratio.

## Results

Dataset characteristics 

The initial dataset consisted of 6625 patients; however, 30 cases were excluded due to blank values in patient variables. The dataset meeting inclusion criteria totaled 6595 patients. Survival data were available for all patients. Table 2 describes the patient characteristics, and Table 3 describes mortality rates in the dataset.

**Table 2:** A summary of baseline patient characteristics including age, gender, primary site, and race.

Characteristics	
Median age (years)	75 (range 2–102)
	N (%)
Gender	
Male	2381 (36.1)
Female	4214 (63.9)
Sites	
Head and neck	1073 (16.3)
Breast	691 (10.5)
Central nervous system	182 (2.8)
Gastrointestinal	900 (13.6)
Genitourinary	1094 (16.6)
Gynecological	525 (8.0)
Hematologic	220 (3.3)
Melanoma	139 (2.1)
Thoracic	1614 (24.5)
Uncategorized	131 (2.0)
Bone	8 (0.12)
Information unclear	18 (0.3)
Race (n=6595)	
Asian	592 (9.0)
Black	1151 (17.5)
Hispanic	1934 (29.3)
Native American	10 (0.1)
White	468 (7.1)
Unknown	2440 (37.0)

**Table 3:** Mortality data of patients included into the dataset (n=6595).

	Alive	Mortality at six months	Mortality at one year	Mortality at three years	Mortality at five years
N (%)	3701 (56.1)	1198 (18.2)	588 (8.9)	837 (12.7)	271 (4.1)
Mean age (years)	74.44	75.64	76.13	75.20	76.53
Gender					
Female (%)	2336 (63)	801 (66.8)	393 (66.8)	516 (61.6)	168 (61.9
Male (%)	1365 (37)	397 (33.2)	195 (33.2)	321 (38.4)	103 (38.1)
Race (%)					
Asian	398 (10.7)	86 (7.18%)	35 (5.95%)	58 (6.93%)	15 (5.54%)
Black	669 (18)	186 (15.5)	72 (12.2)	168 (20.0)	56 (20.6)
Hispanic	992 (26.8)	374 (31.2)	218 (37.0)	266 (31.7)	84 (31.0)
Native American	6 (0.1)	1 (0.08)	1 (0.1)	1 (0.1)	1 (0.3)
White	465 (12.5)	3 (0.2)	0 (0.0)	0 (0.0)	0 (0.0)
Primary disease system (%)					
Bone	2 (0.05)	5 (0.42)	1 (0.17)	0 (0.0)	0 (0.0)
Breast	590 (15.9)	19 (1.5)	11 (1.8)	46 (5.5)	25 (9.2)
CNS	43 (1.1)	76 (6.3)	32 (5.4)	26 (3.1)	5 (1.8)
Gastrointestinal	440 (11.8)	165 (13.7)	99 (16.8)	164 (19.5)	32 (11.8)
Genitourinary	828 (22.3)	83 (6.9)	45 (7.6)	93 (11.1)	45 (16.6)
Gynecologic	331 (8.9)	61 (5.0)	41 (6.9)	64 (7.6)	28 (10.3)
Head and neck	643 (17.3)	159 (13.2)	105 (17.8)	120 (14.3)	46 (16.9)
Hematologic	120 (3.2)	45 (3.7)	15 (2.5)	25 (2.9)	15 (5.5)
Melanoma	76 (2.0)	25 (2.0)	16 (2.7)	18 (2.1)	4 (1.4)
Thoracic	548 (14.8)	531 (44.3)	205 (34.8)	263 (31.4)	67 (24.7)
Uncategorized	74 (2.0)	25 (2.0)	15 (2.5)	15 (1.7)	2 (0.7)

There were 4214 (63.9%) females and 2381 (36.1%) males, with a median age of 75 years (IQR = 8.87 years) and an age range of 2.5-102 years. The cumulative mortality rates within six months, one year, three years, and five years were 18.2%, 27.1%, 39.8%, and 43.9%, respectively. The racial distribution of the dataset was 1934 (29.3%) Hispanic, 1151 (17.5%) African American, 468 (7.1%) White, 592 (9.0%) Asian, and the remainder were not known or “other.” Among the primary sites of malignancy, thoracic cancers were most common, with 1614 (24.5%) cases, followed by genitourinary and gastrointestinal malignancies.

Neural network performance 

**Figure 1:**
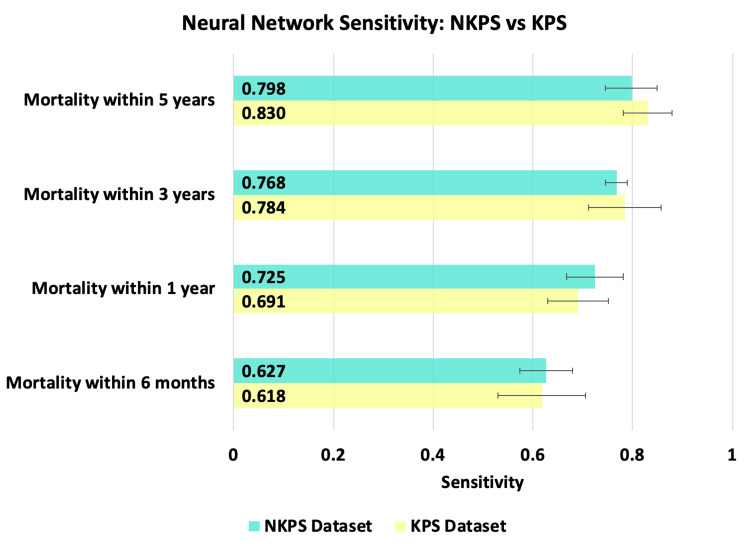
This figure compares the sensitivity of neural networks trained for each of the four mortality time points with the KPS (yellow) and NKPS (green) datasets. Error bars represent standard deviation.

**Figure 2:**
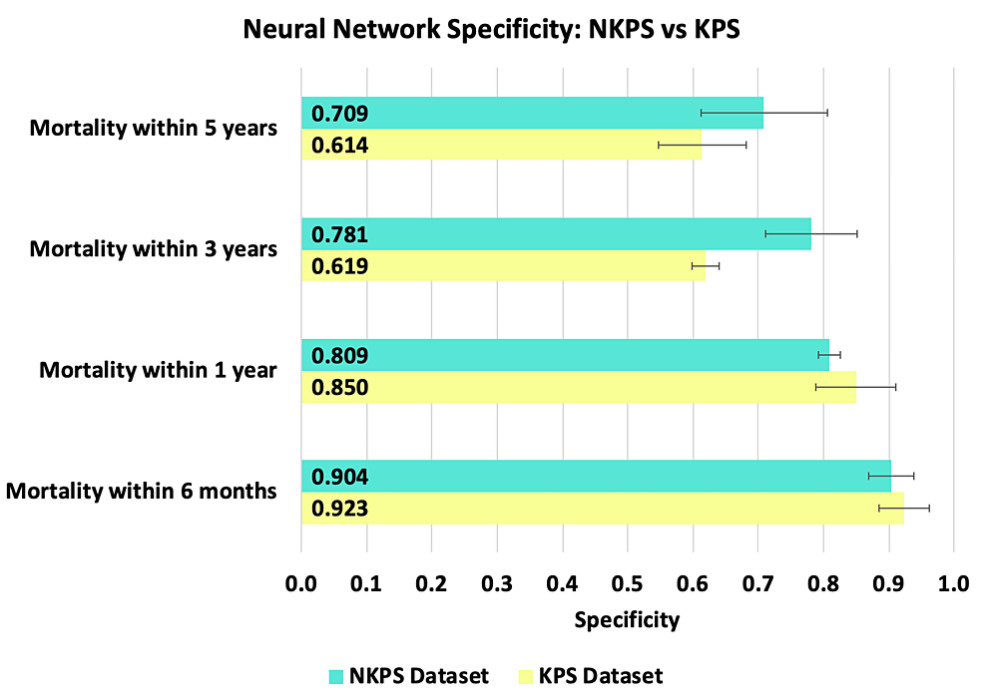
This figure compares the specificity of neural networks trained for each of the four mortality time points with the KPS (yellow) and NKPS (green) datasets. Error bars represent standard deviation.

The best performing ANN for the NKPS dataset demonstrated a sensitivity of 62.71% ± 5.28%, 72.53% ± 5.67%, 76.78% ± 2.17%, and 79.84% ± 5.22% at six months, one year, three years, and five years, respectively. With respect to specificity, the ANN achieved 90.39% ± 3.46%, 80.89% ± 1.59%, 78.09% ± 7.02%, and 70.95% ± 9.73% at six months, one year, three years, and five years, respectively. The best-performing ANN for the KPS dataset demonstrated a sensitivity of 61.76% ± 8.79%, 69.11% ± 6.07%, 78.45% ±7.33%, and 83.00% ± 4.89% at six months, one year, three years, and five years, respectively. With respect to specificity, the ANN achieved 92.35% ± 3.92%, 85.00% ± 6.17%, 61.89% ± 2.13%, and 61.39% ±6.66% at six months, one year, three years, and five years, respectively. The trend over time can be visualized in Figures 1-2.

**Figure 3:**
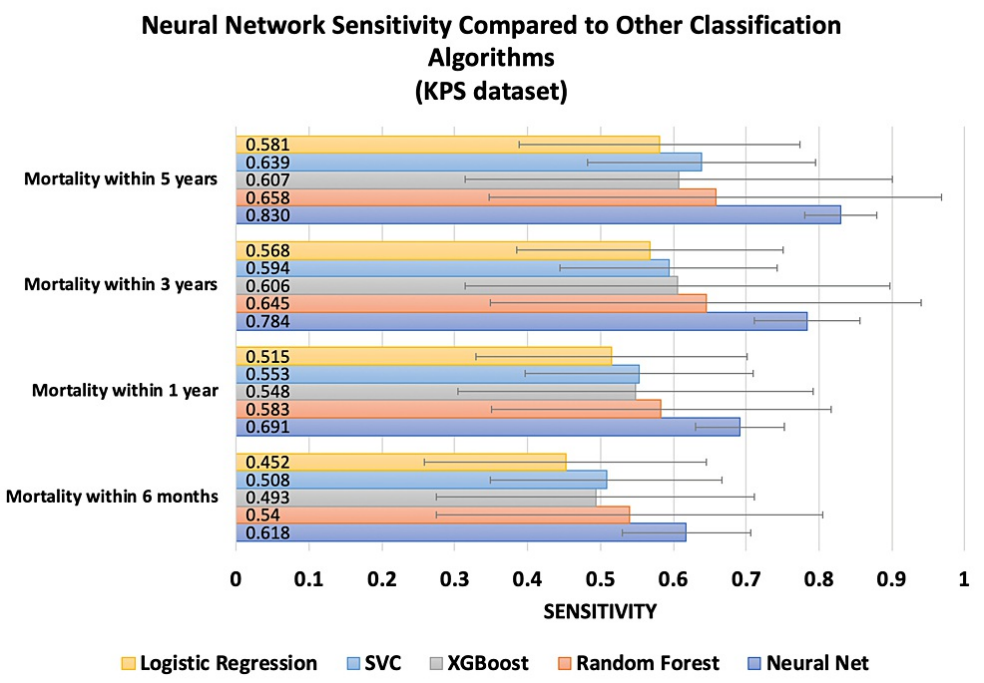
This chart compares the sensitivity of the neural network trained on the KPS dataset against other classification algorithms. Error bars represent standard deviation.

**Figure 4:**
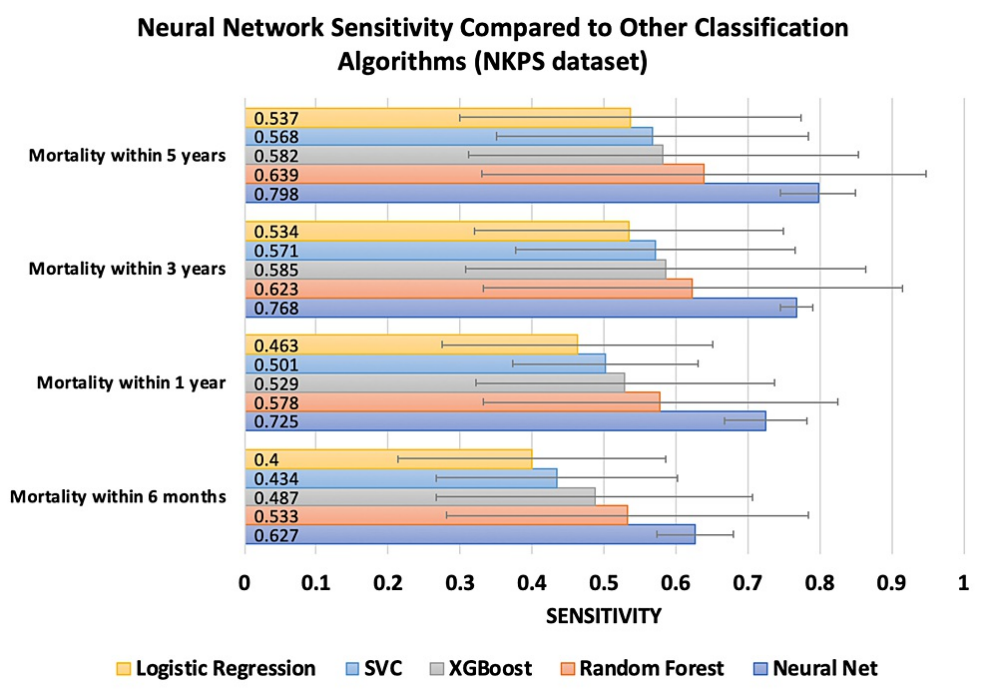
This chart compares the sensitivity of the neural network trained on the NKPS dataset against other classification algorithms. Error bars represent standard deviation.

The sensitivity at all mortality time points for both datasets using ANNs was compared to that of random forest, XGBoost, SVM, and logistic regression algorithms (Figures 3-4).

Tables 4-9 show the full results for neural network models at all mortality time points for both datasets, with respect to sensitivity, specificity, positive predictive value, negative predictive value, and binary accuracy. Tables 10-17 show the performance of comparison models.

**Table 4:** The means and standard deviations of sensitivity and specificity for the individual network configurations with the highest sensitivity for each target variable in the NKPS dataset.

Target variable	Sensitivity	SD	Specificity	SD
Mortality_within_six_months	0.627125	0.052799	0.903927	0.034644
Mortality_within_one_year	0.725324	0.056659	0.808875	0.015871
Mortality_within_three_years	0.767840	0.021721	0.780903	0.070180
Mortality_within_five_years	0.798449	0.052181	0.709483	0.097310

**Table 5:** The means and standard deviations of sensitivity and specificity for the individual network configurations with the highest sensitivity for each target variable in the KPS dataset.

Target variable	Sensitivity	SD	Specificity	SD
Mortality_within_six_months	0.617569	0.087932	0.923528	0.039226
Mortality_within_one_year	0.691093	0.060693	0.849981	0.061708
Mortality_within_three_years	0.784454	0.073281	0.618898	0.021312
Mortality_within_five_years	0.829970	0.048931	0.613884	0.066620

**Table 6:** The means and standard deviations of positive predictive value (PPV) and negative predictive value (NPV) for the individual network configurations with the highest sensitivity for each target variable in the NKPS dataset.

Target variable	PPV	SD	NPV	SD
Mortality_within_six_months	0.600907	0.119304	0.916717	0.017767
Mortality_within_one_year	0.578821	0.067375	0.890591	0.017636
Mortality_within_three_years	0.702895	0.089469	0.833984	0.036096
Mortality_within_five_years	0.689176	0.095160	0.821812	0.024739

**Table 7:** The means and standard deviations of positive predictive value (PPV) and negative predictive value (NPV) for the individual network configurations with the highest sensitivity for each target variable in the KPS dataset.

Target variable	PPV	SD	NPV	SD
Mortality_within_six_months	0.645565	0.096500	0.924072	0.008464
Mortality_within_one_year	0.636857	0.095414	0.887138	0.017602
Mortality_within_three_years	0.621533	0.144608	0.801016	0.026740
Mortality_within_five_years	0.646932	0.072193	0.813368	0.018998

**Table 8:** The mean and standard deviation of binary accuracy for the individual network configurations with the highest sensitivity for each target variable in the NKPS dataset.

Target variable	Binary accuracy	SD
Mortality_within_six_months	0.855497	0.036233
Mortality_within_one_year	0.789841	0.008341
Mortality_within_three_years	0.778620	0.046361
Mortality_within_five_years	0.753904	0.063270

**Table 9:** The mean and standard deviation of binary accuracy for the individual network configurations with the highest sensitivity for each target variable in the KPS dataset.

Target variable	Binary accuracy	SD
Mortality_within_six_months	0.875388	0.031687
Mortality_within_one_year	0.814514	0.039004
Mortality_within_three_years	0.687992	0.119592
Mortality_within_five_years	0.718787	0.035932

**Table 10:** The binary accuracy, sensitivity, specificity, PPV, and NPV of each target variable in the NKPS dataset using Scikit-Learn’s Logistic Regression Classifier. Data are presented as (mean ± standard deviation).

Target variable	Accuracy	Sensitivity	Specificity	PPV	NPV
6-month mortality	(0.832757 ± 0.024858)	(0.400470 ± 0.186079)	(0.928664 ± 0.065404)	(0.626699 ± 0.115164)	(0.877352 ± 0.027736)
1-year mortality	(0.769073 ± 0.029643)	(0.463482 ± 0.188689)	(0.882512 ± 0.097797)	(0.662016 ± 0.118701)	(0.821435 ± 0.037869)
3-year mortality	(0.700538 ± 0.047119)	(0.533650 ± 0.215285)	(0.810675 ± 0.167944)	(0.717973 ± 0.123873)	(0.738532 ± 0.055909)
5-year mortality	(0.674018 ± 0.052384)	(0.536650 ± 0.237321)	(0.781432 ± 0.174428)	(0.699813 ± 0.090135)	(0.702463 ± 0.068471)

**Table 11:** The binary accuracy, sensitivity, specificity, PPV, and NPV of each target variable in the NKPS dataset using Scikit-Learn’s Random Forest Classifier. Data are presented as (mean ± standard deviation).

Target variable	Accuracy	Sensitivity	Specificity	PPV	NPV
6-month mortality	(0.867022 ± 0.030083)	(0.533137 ± 0.250962)	(0.941078 ± 0.055810)	(0.708106 ± 0.146274)	(0.905127 ± 0.045003)
1-year mortality	(0.813346 ± 0.052741)	(0.578245 ± 0.245985)	(0.900603 ± 0.084036)	(0.707212 ± 0.130531)	(0.860805 ± 0.064901)
3-year mortality	(0.717818 ± 0.095264)	(0.623221 ± 0.291191)	(0.780211 ± 0.151089)	(0.648010 ± 0.142925)	(0.790994 ± 0.119717)
5-year mortality	(0.696019 ± 0.103445)	(0.638600 ± 0.309385)	(0.740918 ± 0.165924)	(0.646923 ± 0.131319)	(0.774998 ± 0.147028)

**Table 12:** The binary accuracy, sensitivity, specificity, PPV, and NPV of each target variable in the NKPS dataset using Scikit-Learn’s support vector classifier (SVC) with a linear kernel. Data are presented as (mean ± standard deviation).

Target variable	Accuracy	Sensitivity	Specificity	PPV	NPV
6-month mortality	(0.833516 ± 0.025909)	(0.433862 ± 0.168225)	(0.922179 ± 0.068063)	(0.621949 ± 0.114271)	(0.882639 ± 0.025409)
1-year mortality	(0.771499 ± 0.024619)	(0.501029 ± 0.128682)	(0.871907 ± 0.078977)	(0.628037 ± 0.088899)	(0.827719 ± 0.025264)
3-year mortality	(0.707513 ± 0.044085)	(0.570640 ± 0.193903)	(0.797835 ± 0.157027)	(0.700104 ± 0.106268)	(0.750406 ± 0.053516
5-year mortality	(0.685841 ± 0.042845)	(0.567747 ± 0.216549)	(0.778184 ± 0.155847)	(0.701096 ± 0.079344)	(0.715379 ± 0.067394)

**Table 13:** The binary accuracy, sensitivity, specificity, PPV, and NPV of each target variable in the NKPS dataset using XGBoost. Data are presented as (mean ± standard deviation).

Target variable	Accuracy	Sensitivity	Specificity	PPV	NPV
6-month mortality	(0.828664 ± 0.031134)	(0.487233 ± 0.220399)	(0.904391 ± 0.082643)	(0.592212 ± 0.102863)	(0.892896 ± 0.036766)
1-year mortality	(0.758611 ± 0.039001)	(0.528972 ± 0.208055)	(0.843834 ± 0.103089)	(0.580998 ± 0.091400)	(0.836490 ± 0.050888)
3-year mortality	(0.653690 ± 0.076489)	(0.585090 ± 0.277510)	(0.698892 ± 0.247999)	(0.608151 ± 0.097611)	(0.750523 ± 0.081035)
5-year mortality	(0.672807 ± 0.062456)	(0.581589 ± 0.270724)	(0.744138 ± 0.165698)	(0.653882 ± 0.062865)	(0.724232 ± 0.091678)

**Table 14:** The binary accuracy, sensitivity, specificity, PPV, and NPV of each target variable in the KPS dataset using Scikit-Learn’s Logistic Regression Classifier. Data are presented as (mean ± standard deviation).

Target variable	Accuracy	Sensitivity	Specificity	PPV	NPV
6-month mortality	(0.849853 ± 0.027000)	(0.452301 ± 0.194121)	(0.932008 ± 0.067121)	(0.670624 ± 0.140890)	(0.894496 ± 0.028730)
1-year mortality	(0.781821 ± 0.038241)	(0.514650 ± 0.185721)	(0.878168 ± 0.103170)	(0.662724 ± 0.115342)	(0.839664 ± 0.039860)
3-year mortality	(0.694456 ± 0.062191)	(0.568250 ± 0.182755)	(0.782785 ± 0.191806)	(0.710603 ± 0.130983)	(0.730704 ± 0.044291)
5-year mortality	(0.675594 ± 0.060657)	(0.580745 ± 0.192563)	(0.756886 ± 0.207907)	(0.726468 ± 0.119983)	(0.689991 ± 0.050931)

**Table 15:** The binary accuracy, sensitivity, specificity, PPV, and NPV of each target variable in the KPS dataset using Scikit-Learn’s Random Forest Classifier. Data are presented as (mean ± standard deviation).

Target variable	Accuracy	Sensitivity	Specificity	PPV	NPV
6-month mortality	(0.878253 ± 0.026653)	(0.539952 ± 0.265417)	(0.948142 ± 0.047717)	(0.716826 ± 0.120307)	(0.912925 ± 0.045113)
1-year mortality	(0.816191 ± 0.051928)	(0.583041 ± 0.233384)	(0.900260 ± 0.085078)	(0.706990 ± 0.135313)	(0.864702 ± 0.060390)
3-year mortality	(0.712593 ± 0.088188)	(0.644666 ± 0.295655)	(0.760049 ± 0.213633)	(0.689866 ± 0.120911)	(0.798681 ± 0.123273)
5-year mortality	(0.683942 ± 0.101605)	(0.657867 ± 0.311028)	(0.706391 ± 0.229173)	(0.671817 ± 0.110221)	(0.772077 ± 0.148487)

**Table 16:** The binary accuracy, sensitivity, specificity, PPV, and NPV of each target variable in the KPS dataset using Scikit-Learn’s support vector classifier (SVC) with a linear kernel. Data are presented as (mean ± standard deviation).

Target variable	Accuracy	Sensitivity	Specificity	PPV	NPV
6-month mortality	(0.849855 ± 0.030012)	(0.508049 ± 0.159349)	(0.920484 ± 0.068888)	(0.641753 ± 0.132044)	(0.902883 ± 0.023920)
1-year mortality	(0.771793 ± 0.029985)	(0.553374 ± 0.157479)	(0.850552 ± 0.095683)	(0.611903 ± 0.091664)	(0.845865 ± 0.034382)
3-year mortality	(0.698278 ± 0.065265)	(0.594340 ± 0.148743)	(0.771011 ± 0.186895)	(0.697796 ± 0.124797)	(0.736298 ± 0.034453)
5-year mortality	(0.694217 ± 0.061011)	(0.639234 ± 0.156755)	(0.741374 ± 0.201214)	(0.724350 ± 0.116945)	(0.714662 ± 0.044290)

**Table 17:** The binary accuracy, sensitivity, specificity, PPV, and NPV of each target variable in the KPS dataset using XGBoost. Data are presented as (mean ± standard deviation). Data are presented as (mean ± standard deviation).

Target variable	Accuracy	Sensitivity	Specificity	PPV	NPV
6-month mortality	(0.841499 ± 0.028208)	(0.492655 ± 0.219192)	(0.913570 ± 0.072790)	(0.601638 ± 0.101798)	(0.900849 ± 0.034219)
1-year mortality	(0.734094 ± 0.087883)	(0.547893 ± 0.244319)	(0.801170 ± 0.187702)	(0.577383 ± 0.136363)	(0.843860 ± 0.052098)
3-year mortality	(0.658888 ± 0.078168)	(0.605851 ± 0.291903)	(0.695899 ± 0.249594)	(0.629157 ± 0.097397)	(0.754264 ± 0.092269)
5-year mortality	(0.648613 ± 0.068582)	(0.606625 ± 0.293382)	(0.684669 ± 0.223825)	(0.653761 ± 0.074544)	(0.709464 ± 0.095445)

## Discussion

As the advancements in AI and ML continue to progress, we are in the midst of technology that can potentially revolutionize the field of radiation oncology through enhancing decision-making processes. We summarize a few studies that have utilized AI for outcome prediction in malignancies treated with radiation therapy in Table 18 [14-29].

**Table 18:** Studies that have utilized AI for outcome prediction in malignancies. Pts: patients, RSF: random survival forest, LC: local control, DMFS: distant metastasis-free survival, RC: regional control, CR: complete response, CRT: chemoradiation therapy, PPV: positive predictive value, NPV: negative predictive value, SLNB: sentinel lymph node biopsy, ER: estrogen receptor, LVI: lymphovascular invasion, MoEDL: multi-objective ensemble deep learning, DFS: disease-free survival, Pts: patients, SVM: support vector machine, AUC: area-under-curve, RT: radiation therapy, DVH: dose-volume histogram, ANN: artificial neural network, AI: artificial intelligence, NPC: nasopharyngeal carcinoma, BN: Bayesian network, KNN: k-nearest neighbors, FDG-PET: fluorodeoxyglucose-positron emission tomography, LR: local recurrence, CNN: convolutional neural network, pCR: pathological complete response, MCC: Matthew's correlation coefficient, TCP: tumor control probability.

Study	Cancer site	AI model	Design	Results
Park et al. [14]	Cervical cancer	RSF	93 pts, RSF to predict LC, DMFS, OS, RC	ROC analysis of LC, RC, DMFS, and OS values for the predicted risk were 0.634, 0.796, 0.733, and 0.749 in the validation dataset, respectively.
Shen et al. [15]	Rectal cancer	RSF	RSF using radiomics to predict CR after CRT, 68 features extracted from radiomics	Sensitivity, specificity, PPV, NPV, and accuracy were 81.8%, 97.3%, 81.8%, 97.3%, and 95.3%, respectively.
Madekivi et al. [16]	Breast cancer	XG boost vs logistic regression	Predict nodal stage after SLNB, used variables such as tumor size, histology, multifocality, LVI, ER status, positive nodes number	XG boost able to maintain its discrimination in the validation cohort better than the logistic regression model.
Mortazavi et al. [17]	Whole body	Multilayer perceptron neural network	Model used to predict annual effective radiation exposure dose in 91 radiation workers	Factors such as red cell distribution width, educational degree, non-academic course in radiation protection, working hours per month, department and the number of procedures done per year, and work in the radiology department or not were the most important predictors for annual effective dose.
Wang et al. [18]	Lung cancer	MoEDL	Prediction of DFS. 814 pts that did not achieve DFS and 193 pts that did achieve DFS	MoEDL can perform better than other conventional methods.
Wu et al. [19]	Prostate cancer	SVM	Radiomics study using MRI-based features to predict outcomes. 23 pts included that were treated with carbon-ion therapy	SVM achieved high performance in predicting individualized treatment response of carbon ion therapy, i.e., AUC = 0.88.
Mizutani et al. [20]	Gliomas	SVM	Model used to predict survival after RT, 35 patients, 12 clinical features, and 192 DVHs used	Combined use of clinical and DVH features improved the accuracy of model prediction versus using them separately.
Wang et al. [21]	Lung cancer	ANN	10 pts included to predict spatial and temporal trajectories of lung tumors during RT	ANN predicted tumors on weeks 4, 5, and 6 with a Dice and root mean square surface distance of 0.78 ± 0.22, 0.69 ± 0.24, 0.69 ± 0.26, and 2.1 ± 1.1 mm, 2.3 ± 0.8 mm, 2.6 ± 1.4 mm respectively.
Zhao et al. [22]	NPC	SVM	123 patients using radiomics and clinical data to predict response to induction chemotherapy	Radiomics nomogram with clinical data worked better than clinical nomogram alone (C-index in the validation cohort, 0.863 vs 0.549; p)
Shayesteh et al. [23]	Rectal cancer	Models tested included SVM, BN, ANN, and KNN classifiers	98 patients included to predict response to neoadjuvant CRT	In the ensemble machine learning models, the best result was for the SVM, ANN, BN, and KNN classifiers, with an accuracy of 92.8% in testing and 90% in the validation set, respectively.
Shen et al. [24]	Cervix cancer	k-fold cross-validation	142 patients using deep learning to predict FDG-PET response to CRT	Regarding LR, the sensitivity, specificity, PPV, NPV, and accuracy were 71%, 93%, 63%, 95%, and 89%, respectively, while the corresponding values for distant metastasis were 77%, 90%, 63%, 95%, and 87%, respectively.
Tian et al. [25]	Gynecological cancers undergoing Brachytherapy	SVM	35 patients were included in the study to predict fistula formation. Sequential backward feature selection and sequential floating backward feature selection methods were used to determine optimal feature sets	The model achieved a 97.1% sensitivity, and 88.5% specificity.
Shi et al. [26]	Rectal cancer	CNN	51 patients, prediction of response to pre-operative CRT using radiomics (pCR).	Combining tumor region of interest and radiomics achieved an AUC of 0.80 for pre-treatment, 0.82 for mid-RT, and 0.86 for both MRIs together.
Li et al. [27]	Lung cancer	Logistic regression	For prediction of local recurrence, logistic regression and repeated stratified five-fold cross-validation (CV) using PET-CT data.	For predicting overall relapse, the best classifier found had a mean MCC of 0.29 and was composed of a single feature: the volume greater than 0.5 times the maximum SUV.
Larroza et al. [28]	Brain cancer	SVM	Prediction to differentiate between radiation necrosis versus brain metastasis based on MRI, 115 lesions.	High classification accuracy (AUC > 0.9) was obtained using texture features and a support vector machine classifier.
Klement et al. [29]	Lung cancer	SVM	Predicting TCP in 399 patients.	Sensitivity and specificity were 67.0% ± 0.5% and 78.7% ± 0.3%, respectively.

Predictive models can be created by applying advanced algorithms and analyzing large datasets to assist in personalized treatment planning and decision-making. With hyperparameter grid-searching, network configurations optimizing the algorithm for sensitivity were found for six-month, one-year, three-year, and five-year mortality. The ANN was able to achieve a higher sensitivity than other machine learning algorithms across all output target variables, demonstrating the utility of an ANN model for mortality prediction on the provided dataset.

The implemented ANNs were successfully able to perform binary prediction of mortality with an accuracy greater than random chance. The neural networks consistently demonstrated higher average sensitivities and lower standard deviations than all other models across both datasets and all mortality variables. This is an indication that the neural networks were able to extrapolate patterns and heuristics from the variables present in the clinical dataset. Interestingly, the neural networks trained on the NKPS dataset appeared to perform with more precision in terms of sensitivity and specificity compared to the results from the KPS dataset. We believe that this result may be attributable to the removal of 36.33% of the total cancer cases in the KPS dataset, leading to less reliable learning.

The neural network was able to achieve higher sensitivity for mortality prediction over a longer time frame compared to a shorter time frame. A plausible reason this occurred was the increased number of positive cases per mortality variable over a five-year time span. Aside from the imbalance of positive cases, the prediction of mortality in a shorter time frame is more difficult because there is a greater influence of random chance. Prediction over a longer time frame is a more tractable problem for the algorithm since the influence of random chance is reduced while the impact of the prognostic variables used as inputs takes precedence. In contrast to sensitivity, specificity was seen to decrease over time due to the neural network being more prone to making positive predictions with a lower detection threshold. As the “excitability” of the network increases, more type 1 errors occur, thus lowering the specificity. This is typical of the trade-off between sensitivity and specificity.

Binary accuracy was seen to decrease with each mortality time point, which can be explained in the context of data dynamics. As time increases, the mortality proportion of the dataset naturally increases, which leads to increased sensitivity and decreased specificity, as noted before. Additionally, there is increased uncertainty due to a greater number of variables or events that could affect mortality over time, such as comorbid conditions. Therefore, if the model does not perfectly capture the relevant temporal dynamics of the dataset, accuracy will decrease with time. We interpret binary accuracy as a less useful evaluator of neural network performance because of its dependence on target variable prevalence. For example, if there were zero cases of mortality at six months, the network could achieve a binary accuracy of 100% with 100% specificity by outputting only negative predictions with 0% sensitivity. Similar to binary accuracy, PPV and NPV are less useful evaluators of performance because of their similar dependence on target variable prevalence. Therefore, PPV and NPV, in addition to binary accuracy, are subject to the distribution of the data we are using to train the model.

The ratio of positive and negative samples differs greatly amongst the mortality variables at different follow-up dates, so it would be preferable to use a metric that can be consistently applied to all target variables. Sensitivity is intrinsic to the classifier system and cannot be affected by prevalence. However, it is significant to note that, in practical use, PPV is the most important metric in assessing the probability of mortality in a patient given a positive output from any model.

The clearest limitation of this study was the relatively small (N = 6595) dataset of cancer cases. Although this is considered a large medical cohort, especially when compared to other published studies using ML, it is small for machine learning. With more training samples, the networks could possibly develop a more nuanced understanding of the variables to achieve higher sensitivity and specificity. Another limitation specific to this dataset was the class imbalance inherent to mortality outcomes. There were significantly more survivors at six months compared to deceased individuals, which is not ideal for training a classification algorithm. A super-sampling technique such as the synthetic minority oversampling technique (SMOTE) may be employed in the future to achieve a balanced dataset to feed into the machine learning algorithm at training time and potentially improve performance [30]. An internally obvious limitation was the lack of powerful hardware to run this study. Early attempts were made to run the grid search across hundreds of parameters (in excess of 700), but after several days of training, it became apparent that such program run-times were unacceptable. If greater computing power were available, a wider variety of network architectures could be explored; for instance, deeper networks with three or more layers.

## Conclusions

The use of ANNs resulted in a successful mortality prediction model and was shown to outperform other machine learning algorithms with greater sensitivity and specificity. The unique features of our study, when compared to similar studies, include a relatively large and longitudinal sample size as we are a multisite institution composed of both academic and community medical centers, a broad set of pathological diagnoses, and high regional diversity. Moving forward, we believe there is room for improvement in our current model through the refinement of prognostic variables that were used as network inputs, such as the inclusion of radiomics (imaging data in the form of CT or MRI images). A convolutional neural network architecture that incorporates both prognostic and imaging data for each patient could theoretically increase performance by a vast margin. Future projects involving imaging data will require significantly more computing power than what was available in this study.

## Appendices

**Table 19:** Enumeration of values for categorical variables.

Variable	Possible Values
Sex	['Female', 'Male']
Race	['Asian', 'Black', 'Hispanic', 'Native American', 'White']
Cancer_site	['Abdomen', 'Anal', 'Appendix', 'Basal cell skin', 'Bile duct', 'Bladder', 'Bone', 'Brain', 'Breast', 'Bronchus', 'Cervix', 'Colon', 'Ear', 'Endometrium', 'Esophagus', 'Eye', 'Fallopian tube', 'Gastrointestinal', 'Genitourinary', 'Gynecologic', 'Head & neck', 'Heart', 'Hematologic', "Hodgkin's lymphoma", 'Hypopharynx', 'Intestine', 'Kidney', 'Larynx', 'Leukemia', 'Limbs', 'Lip', 'Liver', 'Lungs', 'Lymphoma', 'Mediastinum', 'Melanoma', 'Meninges', 'Merkel cell skin', 'Myeloma', 'Nasal cavity', 'Nasopharynx', "Non-Hodgkin's lymphoma", 'Oral cavity', 'Oropharynx', 'Ovary', 'Pancreas', 'Paraganglioma', 'Pelvis', 'Penis', 'Pharynx', 'Pituitary gland', 'Pleura', 'Prostate', 'Rectum', 'Salivary glands', 'Sarcoma', 'Scalp', 'Sinuses', 'Skin', 'Soft tissue sarcoma', 'Spine', 'Stomach', 'Testis', 'Thorax', 'Thymus', 'Thyroid gland', 'Tonsils', 'Trachea', 'Ureter', 'Urethra', 'Uterus', 'Vagina', 'Vulva']
Treatment_site	['Abdomen', 'Adrenal gland', 'Anal', 'Back', 'Basal cell skin', 'Bile duct', 'Bladder', 'Bone', 'Brain', 'Breast', 'Bronchus', 'Buttock', 'Cervix', 'Colon', 'Cranium', 'Ear', 'Endometrium', 'Esophagus', 'Eye', 'Fallopian tube', 'Head & neck', 'Heart', 'Hypopharynx', 'Intestine', 'Kidney', 'Larynx', 'Limbs', 'Lip', 'Liver', 'Lungs', 'Lymph nodes', 'Lymphoma', 'Mediastinum', 'Melanoma', 'Meninges', 'Myeloma', 'Nasal cavity', 'Nasopharynx', "Non-Hodgkin's lymphoma", 'Nose', 'Oral cavity', 'Oropharynx', 'Ovary', 'Pancreas', 'Paraganglioma', 'Pelvis', 'Penis', 'Pharynx', 'Pituitary gland', 'Pleura', 'Prostate', 'Rectum', 'Salivary glands', 'Scalp', 'Sinuses', 'Skin', 'Soft tissue sarcoma', 'Spine', 'Stomach', 'Testis', 'Thorax', 'Thymus', 'Thyroid gland', 'Tonsils', 'Total body', 'Trachea', 'Ureter', 'Urethra', 'Uterus', 'Vagina', 'Vulva']
Stage	['I', 'II', 'III', 'IV']
Overall_stage	['!AE', '0', '0is', '1', '1E (rec)', '4', '88', 'ERROR', 'Extensive', 'I', 'IA', 'IA2', 'IA3', 'IAE', 'IB', 'IB1', 'IB2', 'IBNOS', 'IC', 'IE', 'IEA', 'II', 'IIA', 'IIA1', 'IIAE', 'IIB', 'IIC', 'IIE', 'IIEA', 'III', 'IIIA', 'IIIB', 'IIIC', 'IIIC1', 'IIIC2', 'IIINOS', 'IIIS', 'IINOS', 'IIS', 'IIa', 'INOS', 'IV', 'IVA', 'IVB', 'IVBE', 'IVC', 'IVNOS', 'IVa', 'Ie', 'Iv', 'Limited', 'Limited st', 'Metastatic', 'N/a', 'N?A', 'OCCULT', 'Rec', 'Recurrence', 'Recurrent', 'Solitary', 'UNK', 'X']
Cancer_histology	['Acinar cell carcinoma', 'Acinar cell tumor', 'Acute leukemia, NOS', 'Acute myelofibrosis', 'Acute myeloid leukemia, NOS', 'Adenocarcinoma w/ neuroendocrine differentiation', 'Adenocarcinoma, NOS', 'Adenocarcinoma, endocervical type', 'Adenocarcinoma, intestinal type (T-151.__)', 'Adenocarcinoma, metastatic, NOS', 'Adenocarcinoma-in-situ', 'Adenoid cystic carcinoma', 'Adenolymphoma (T-142.__)', 'Adenoma, NOS', 'Adenosarcoma', 'Adenosquamous carcinoma', 'Alveolar rhabdomyosarcoma', 'Angiomyosarcoma', 'Apocrine adenocarcinoma', 'Astrocytoma, NOS (T-191.__)', 'Astrocytoma, anaplastic type (T-191.__)', 'Atypical carcinoid tumor', 'Atypical fibroxanthoma', 'Basal cell carcinoma, nodular', 'Basaloid squamous cell carcinoma', 'Basel cell carcinoma, NOS (T-173.__)', 'Carcinoid tumor, malignant', 'Carcinoma in pleomorphic adenoma', 'Carcinoma, NOS', 'Carcinoma, anaplastic type, NOS', 'Carcinoma, metastatic, NOS', 'Carcinoma, undifferentiated type, NOS', 'Carcinoma-in-situ, NOS', 'Carcinosarcoma, NOS', 'Chondrosarcoma, NOS (T-170.__)', 'Chordoma', 'Chromophobe adenoma (T-194.3)', 'Chronic leukemia, NOS', 'Chronic lymphocytic leukemia', 'Clear cell adenocarcinoma, NOS', 'Clear cell tumor, NOS', 'Cloacogenic carcinoma', 'Comedocarcinoma, non-infiltrating (T-174.__)', 'Cribriform carcinoma in situ', 'Cystadenocarcinoma, NOS', 'Cystadenoma, NOS', 'Dedifferentiated liposarcoma', 'Desmoplastic melanoma, malignant', 'Ductal carcinoma in situ, solid type', 'Endometrioid adenocarcinoma, ciliated cell variant', 'Endometrioid adenoma, NOS', 'Endometrioid adenoma, borderline malignancy', 'Endometrioid carcinoma', 'Epithelioid leiomyosarcoma', 'Esthesioneuroblastoma (T-160.__)', 'Fibromyxosarcoma', 'Fibrosarcoma, NOS', 'Fibrous histiocytoma, NOS', 'Fibrous histiocytoma, malignant', 'Fibrous meningioma (T-192.__)', 'Follicular adenocarcinoma, NOS (T-193.9)', 'Giant cell sarcoma (except Bone M-9250/3)', 'Glioblastoma with sarcomatous component (T-191.__)', 'Glioblastoma, NOS (T-191.__)', 'Glioma, malignant (T-191.__)', 'Gliomatosis cerebri (T-191.__)', 'Glomangiosarcoma', 'Hemangiopericytoma, NOS', 'Hemangiosarcoma', 'Hepatocellular carcinoma, NOS (T-155.0)', 'Hodgkin lymphoma, lymphocyte-rich', "Hodgkin's disease, NOS", "Hodgkin's disease, lymphocytic predominance, diffuse", "Hodgkin's disease, nodular sclerosis, NOS", 'Infiltrating duct and lobular carcinoma', 'Infiltrating duct carcinoma (T-174.__)', 'Infiltrating duct mixed w/ other types of carcinoma', 'Infiltrating ductal carcinoma', 'Infiltrating lobular mixed w/ other types of carcinoma', 'Inflammatory carcinoma (T-174.__)', 'Intracystic carcinoma, NOS', 'Intraductal carcinoma and lobular carcinoma in situ', 'Intraductal carcinoma, non-infiltrating, NOS', 'Intraductal papillary adenocarcinoma with invasion', 'Intraductal papillary-mucinous carcinoma, invasive', "Kaposi's sarcome", 'Large cell (Ki-1+) lymphoma', 'Large cell carcinoma, NOS', 'Large cell neuroendocrine carcinoma', 'Leiomyosarcoma, NOS', 'Lipoma, atypical', 'Liposarcoma, NOS', 'Liprosarcoma, well differentiated type', 'Lobular carcinoma, NOS (T-174.__)', 'Lymphoepithelial carcinoma', 'Lymphoma, NK/T-cell, nasal & nasal-type', 'Lymphoma, marginal zone B-cell, NOS', 'Lymphoma, mediastinal large B-cell', 'Malignant lymphoma, NOS', 'Malignant lymphoma, diffuse, NOS', 'Malignant lymphoma, follicular, NOS', 'Malignant lymphoma, large cell, cleaved, diffuse', 'Malignant lymphoma, large cell, diffuse, NOS', 'Malignant lymphoma, large cell, follicular, NOS', 'Malignant lymphoma, lymphocytic, intermediate diff, diffuse', 'Malignant lymphoma, mixed small and large cell, diffuse', "Malignant lymphoma, non-Hodgkin's type", 'Malignant lymphoma, small cleaved cell, diffuse', 'Malignant melanoma, NOS', 'Malignant tumor, giant cell type', 'Malignant tumor, small cell type', 'Marginal zone lymphoma, NOS', 'Medullary carcinoma with amyloid stroma', 'Meningioma, NOS (T-192.__)', 'Meningioma, atypical', 'Merkel cell carcinoma', 'Mesodermal mixed tumor', 'Mesothelioma, malignant', 'Metaplastic carcinoma, NOS', 'Metastatic signet ring cell carcinoma', 'Mixed tumor, malignant, NOS', 'Mucinous adenocarcinoma', 'Mucinous adenocarcinoma, endocervical type', 'Mucoepidermoid carcinoma', 'Mucosal lentiginous melanoma', 'Mucosal-associated lymphoid tissue (MALT) lymphoma', 'Muellerian mixed tumor', 'Multiple myeloma', 'Mycosis fungoides', 'Myeloid sarcoma', 'Myxoid leiomyosarcoma', 'Myxoid liposarcoma', 'Neoplasm, benign', 'Neoplasm, malignant', 'Neoplasm, malignant, uncertain whether primary or metastatic', 'Neoplasm, metastatic', 'Neoplasm, uncertain whether benign or malignant', 'Neurilemmoma, malignant', 'Neuroblastoma, NOS', 'Neuroendocrine carcinoma', 'Neurofibrosarcoma', 'Nodular melanoma', 'Non-infiltrating intraductal papillary adenocarcinoma', 'Non-small cell carcinoma', 'Oat cell carcinoma (T.162.__)', 'Odontogenic tumor, malignant (T-170.__)', 'Olfactory neurogenic tumor', 'Osteosarcoma, NOS (T-170.__)', "Paget's disease, extramammary (except Paget's disease of bone)", 'Papillary adenocarcinoma, NOS', 'Papillary and follicular adenocarcinoma (T-193.9)', 'Papillary carcinoma, NOS', 'Papillary carcinoma, columnar cell', 'Papillary carcinoma-in-situ', 'Papillary cystadenocarcinoma, NOS (T-183.0)', 'Papillary serous cystadenocarcinoma (T-183.0)', 'Papillary squamous cell carcinoma', 'Papillary squamous cell carcinoma, non-invasive', 'Papillary transitional cell carcinoma', 'Papilloma, NOS (except Papilloma of urinary bladder M-8120/1)', 'Peripheral T-cell lymphoma, NOS', 'Pinealoma (T-194.4)', 'Pituitary adenoma, NOS', 'Plasmacytoma, NOS', 'Plasmacytoma, extramedullary (not in bone)', 'Pleomorphic carcinoma', 'Renal cell carcinoma (T-189.0)', 'Rhabdomysarcoma, NOS', 'Sarcoma, NOS', 'Sebaceous adenocarcinoma (T-173.__)', 'Seminoma, NOS (T-186.__)', 'Serous cystadenocarcinoma, NOS (T-183.0)', 'Serous surface papillary carcinoma (T-183.0)', 'Serous surface papilloma, NOS (T-183.0)', 'Signet ring cell carcinoma', 'Skin appendage adenoma (T-173.__)', 'Small cell carcinoma, NOS', 'Solitary fibrous tumor, malignant', 'Spindle cell carcinoma', 'Spindle cell melanoma, NOS', 'Spindle cell sarcoma', 'Squamous cell carcinoma, NOS', 'Squamous cell carcinoma, keratinizing type, NOS', 'Squamous cell carcinoma, large cell, non-keratinizing type', 'Squamous cell carcinoma, metastatic, NOS', 'Squamous cell carcinoma, spindle cell type', 'Squamous cell carcinoma-in-situ, NOS', 'Squamous cell papilloma', 'Synovial sarcoma, NOS', 'Thymic carcinoma, NOS', 'Thymoma, malignant (T-164.0)', 'Thymoma, type B3, malignant', 'Transitional cell carcinoma, NOS', 'Transitional cell carcinoma, spindle cell type', 'Tumor cells, malignant', 'Tumor, malignant, clear cell type', 'Undifferentiated sarcoma', 'Urothelial papilloma', 'Verrucous carcinoma, NOS']
Main_diagnosis_type	['Primary malignant neoplasm', 'Secondary malignant neoplasm']
Primary_disease_system	['Bone', 'Breast', 'Central nervous system', 'Gastrointestinal', 'Genitourinary', 'Gynecologic', 'Head & neck', 'Hematologic', 'Melanoma/sarcoma', 'Thoracic', 'Uncategorized']
Treatment_intent	['Benign', 'Curative', 'De-Bulking', 'Induction-Primary', 'Palliative', 'Primary-Neoadjuvant', 'True Negative', 'pre-op']
Treatment_disease_system	['Bone', 'Breast', 'Central nervous system', 'Gastrointestinal', 'Genitourinary', 'Gynecologic', 'Head & neck', 'Hematologic', 'Melanoma/sarcoma', 'Thoracic', 'Uncategorized']
Religion	['Adventist', 'Baptist', 'Christian (non-Catholic, non-specific)', 'Episcopalian', 'Evangelical Covenant', 'Hinduism', 'Islam', "Jehovah's Witnesses", 'Judaism', 'Lutheran', 'Methodist', 'Orthodox', 'Presbyterian', 'Protestant', 'Roman Catholic Church', 'Zen Buddhism']
Marital_status	['Divorced', 'Married', 'Single', 'Widowed']
Stage_t	['0', '1', '1a', '1b', '1c', '1e', '2', '2a', '2b', '2c', '3', '3a', '3b', '4', '4a', '4b', 'I', 'IA', 'IIB', 'Is', 'T0', 'T1', 'T1NOS', 'T1a', 'T1b', 'T1b1', 'T1b2', 'T1bNOS', 'T1c', 'T1mi', 'T1mic', 'T2', 'T2NOS', 'T2a', 'T2a1', 'T2a2', 'T2b', 'T2c', 'T3', 'T3NOS', 'T3a', 'T3b', 'T3c', 'T4', 'T4NOS', 'T4a', 'T4b', 'T4d', 'TX', 'Tis', 'Tis (DCIS)', 'X']
Stage_n	['0', '1', '1a', '1b', '1c', '2', '2a', '2b', '2c', '3', '3a', '3b', '3c', 'N0', 'N0(i+)', 'N0(i-)', 'N1', 'N1NOS', 'N1a', 'N1b', 'N1c', 'N1mi', 'N2', 'N2NOS', 'N2a', 'N2b', 'N2c', 'N3', 'N3NOS', 'N3a', 'N3b', 'N3c', 'NX', 'Nx', 'X']
Stage_m	['0', '1', '1IV', '1a', '1b', '1c', 'I', 'M0', 'M1', 'M1NOS', 'M1a', 'M1b', 'M1c', 'MX', 'Mx', 'O', 'X']
Treatment_energy	['Cobalt-60', 'Electrons', 'Iodine-125', 'Iridium-192', 'Palladium-103', 'Photons', 'Photons + Electrons', 'Radium-223', 'Yttrium-90']
Treatment_modality	['2D RT / 3D CRT', 'Brachy', 'Gamma Knife', 'IMRT', 'SBRT', 'VMAT']
CB_dermatitis_radiation	['No', 'Yes']
